# Mild Anastomotic Stenosis in Patient-Specific CABG Model May Enhance Graft Patency: A New Hypothesis

**DOI:** 10.1371/journal.pone.0073769

**Published:** 2013-09-13

**Authors:** Yunlong Huo, Tong Luo, Julius M. Guccione, Shawn D. Teague, Wenchang Tan, José A. Navia, Ghassan S. Kassab

**Affiliations:** 1 Mechanics and Engineering Science, College of Engineering, Peking University, Peking, China; 2 State Key Laboratory for Turbulence and Complex Systems, College of Engineering, Peking University, Peking, China; 3 Biomedical Engineering, Indiana University-Purdue University Indianapolis, Indiana, United States of America; 4 Surgery, Indiana University-Purdue University, Indianapolis, Indiana, United States of America; 5 Cellular and Integrative Physiology, Indiana University-Purdue University, Indianapolis, Indiana, United States of America; 6 Surgery, University of California San Francisco, San Francisco, California, United States of America; 7 Bioengineering, University of California San Francisco, San Francisco, California, United States of America; 8 Cardiac Surgery, Austral University, Buenos Aires, Argentina; University of Western Australia, Australia

## Abstract

It is well known that flow patterns at the anastomosis of coronary artery bypass graft (CABG) are complex and may affect the long-term patency. Various attempts at optimal designs of anastomosis have not improved long-term patency. Here, we hypothesize that mild anastomotic stenosis (area stenosis of about 40–60%) may be adaptive to enhance the hemodynamic conditions, which may contribute to slower progression of atherosclerosis. We further hypothesize that proximal/distal sites to the stenosis have converse changes that may be a risk factor for the diffuse expansion of atherosclerosis from the site of stenosis. Twelve (12) patient-specific models with various stenotic degrees were extracted from computed tomography images using a validated segmentation software package. A 3-D finite element model was used to compute flow patterns including wall shear stress (WSS) and its spatial and temporal gradients (WSS gradient, WSSG, and oscillatory shear index, OSI). The flow simulations showed that mild anastomotic stenosis significantly increased WSS (>15 dynes⋅cm^−2^) and decreased OSI (<0.02) to result in a more uniform distribution of hemodynamic parameters inside anastomosis albeit proximal/distal sites to the stenosis have a decrease of WSS (<4 dynes⋅cm^−2^). These findings have significant implications for graft adaptation and long-term patency.

## Introduction

Coronary artery bypass graft (CABG) is a highly-effective therapeutic treatment to relieve symptoms of ischemic heart diseases [1], which is often comprised of internal mammary artery graft (IMAG) or saphenous vein graft (SVG) [2], [3]. The latter appears to have a relatively shorter life span (50% fail at 10–15 years after CABG operation) [2], [4]. The anastomosis between SVG and coronary artery is the frequent site for graft failure [5], which is related to abnormal flow patterns [6]. Although the anastomotic geometry of SVG can significantly affect hemodynamics, the long-term patency has not been completely satisfactory even with an optimized graft anastomosis [7]. Therefore, there is a need to understand the longitudinal hemodynamics at anastomosis postoperatively.

Computational fluid dynamics (CFD) is a common method to determine hemodynamic risk factors (e.g., low wall shear stress-WSS, high oscillatory shear index-OSI, high WSS gradient-WSSG, long residence time caused by flow stagnation, etc.) for atherosclerosis [8]–[13]. This method was used to investigate abnormal flow patterns in grafts by many researchers [14]–[18]. These studies mainly focused on the relation between intimal hyperplasia and hemodynamic parameters. In general, intimal hyperplasia contributes to the short-term (<1 year) graft stenosis while atherosclerosis affects the long-term outcomes. Currently, there is a lack of hemodynamic studies on long-term SVG patency. Hence, the objective of this study was to determine patient-specific hemodynamics at end-to-side anastomoses of SVG and coronary artery with various degrees of stenoses at long term. Here, we hypothesize that mild anastomotic stenosis (area stenosis of 40–60%) may be an adaptive process to improve hemodynamic conditions to enhance the long-term SVG patency (≥10 years) [19], [20]. Conversely, the proximal/distal sites to the stenosis have adverse hemodynamic changes that may contribute to the expansion of atherosclerosis.

In the present study, a transient 3-D finite element (FE) model was used to solve the continuity and Navier-Stokes equations with the measured inlet flow boundary condition and the stress-free outlet boundary condition in geometrical models of SVG and coronary arteries obtained from patient computer tomography (CT) images. The hemodynamic parameters including WSS, OSI, and WSSG were computed at anastomoses based on the computed flow field. These parameters were also computed in four idealized geometric models to mimic the potential effects of various SVG anastomoses on flow patterns, which were compared with those in patient-specific SVG models to show the effects of mild stenosis on local flow dynamics. The significance, implication and limitation of flow simulations are discussed in relation to the long-term SVG patency.

## Materials and Methods

### Study Design

**Figure 1:**
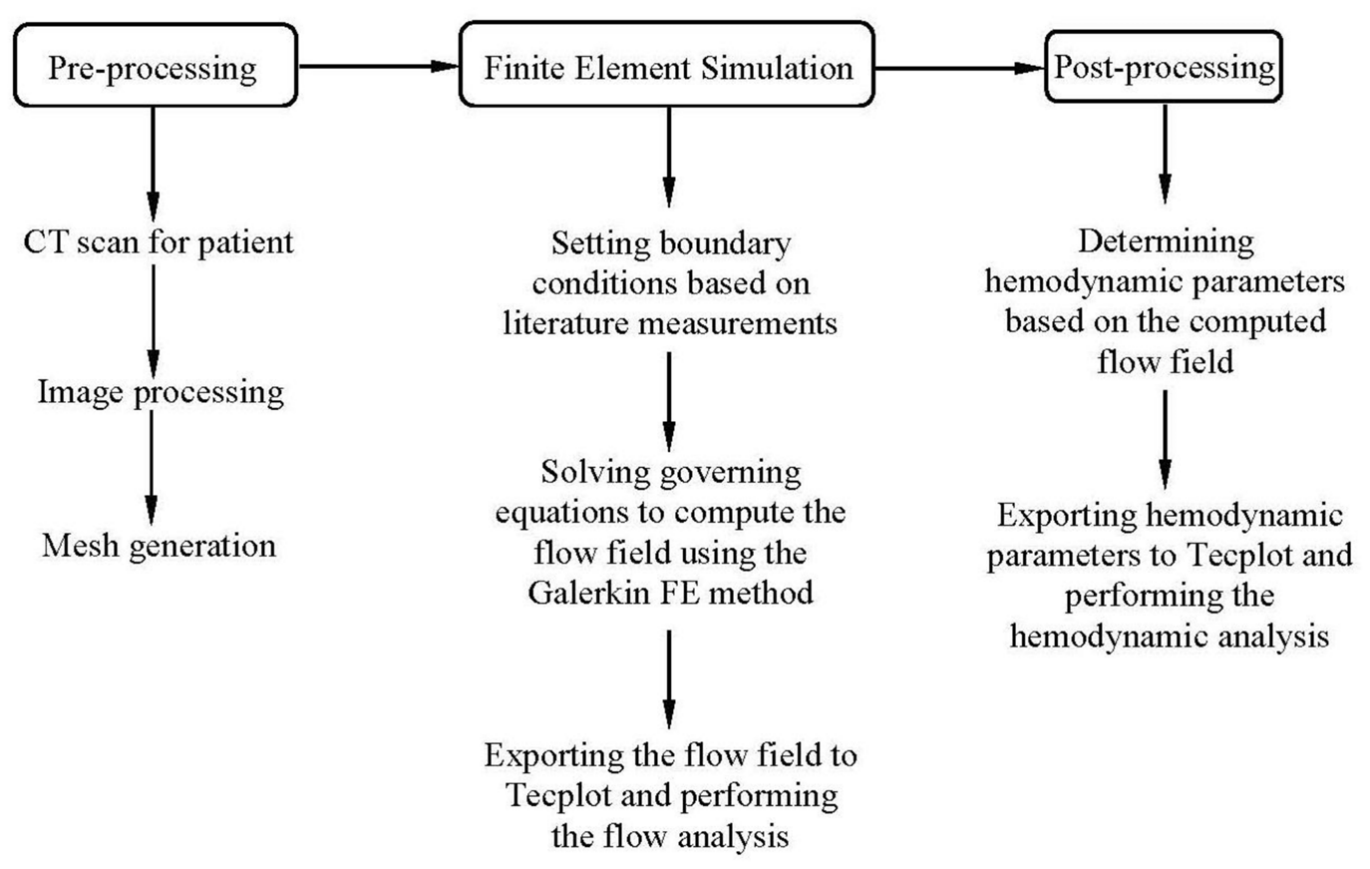
A flow chart depicting imaging acquisition, generation of geometrical model, FE simulation and hemodynamic analysis.

The purpose of this retrospective study was to investigate hemodynamic changes near anastomoses of SVG and coronary artery that had stenoses of various degrees. Twelve human subjects underwent CT angiography (CTA) of coronary arteries for >1 year after the SVG connected the aorta to the coronary artery with end-to-side anastomosis. The retrospective study was approved by the Institutional Review Board (IRB) for Indiana University and the human subjects provided written informed consent to participate in this study. A flow chart in Figure 1 shows the patient-specific imaging, generation of geometrical model, FE simulations and hemodynamic analysis.

### Imaging Acquisition

Prior to the imaging acquisition, patients were given repeated doses of intravenous metoprolol of 5 mg every 5 minutes until heart rate was ≤65 bpm or a maximum dose of 15 mg was given. All patients received sublingual nitroglycerin tablet (0.4 mg) 3–5 minutes before CT examination.

All studies were performed on a dual-source CT scanner (Siemens Definition, Forchheim Germany) similar to a previous study [21]. After an initial survey scan, a retrospectively gated contrast-enhanced scan was obtained using 80 ml of iodinated contrast (Iopromide-Ultravist 370, Bayer Healthcare, Morristown USA) injected through an antecubital vein at 5 ml/s followed by 50 ml of normal saline at the same rate. The scan parameters were: 2×64×0.6 mm collimation, tube voltage –120 kV; tube current – average 620 mAs adjusted to body size; gantry rotation time –330 msec; pitch –0.2–0.43 depending on heart rate. The simultaneous acquisition of multi-parallel cross sections enabled the imaging of SVG and coronary artery in a single breath hold. Images were reconstructed with a slice thickness/increment of 0.7/0.4 mm with B26f at temporal resolution of 83 msec (half-scan). The initial data window was positioned at 70% of the R-R interval, with additional data sets reconstructed at ±5% intervals to compensate for motion artifacts in coronary arteries if necessary [21].

### Imaging Analysis and Geometrical Models

**Figure 2:**
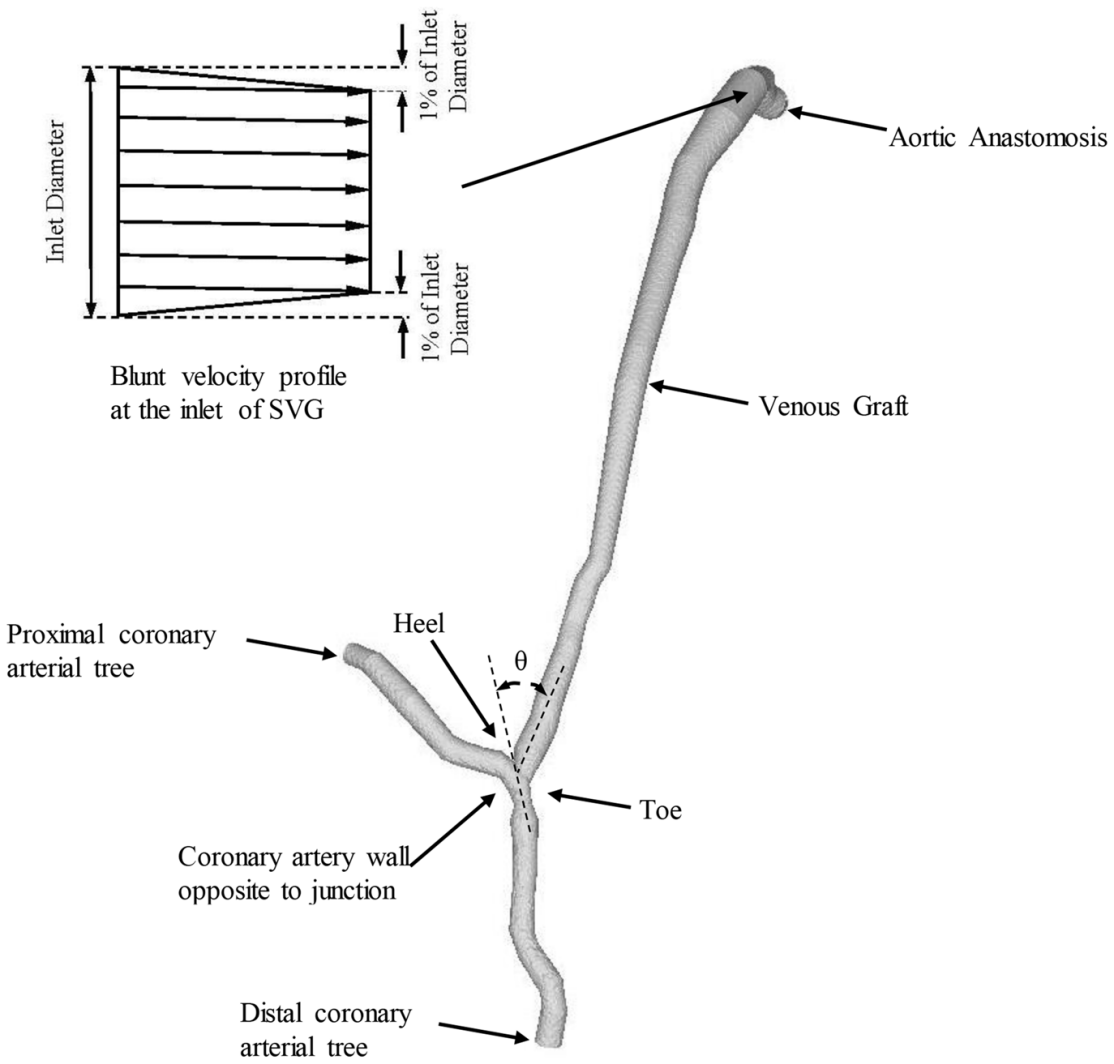
End-to-side SVG obtained from patient CTA, where θ is the graft angle between SVG and coronary artery. A blunt velocity profile was used in the FE simulation.

As shown in Fig. 2, morphometric data of SVG and coronary artery were extracted from CTA images by a validated software package [22], [23]. The algorithm has been validated by optical measurements with a RMS error of 0.16 mm (<10% of the mean value) and an average deviation of 0.13 mm. The morphometric data were imported to ANSYS software to generate FE meshes, similar to a previous study [24].

### 3-D FE Model and Hemodynamic Analysis

The governing equations are formulated for SVG and coronary artery, each vessel of which is assumed to be rigid and to have impermeable wall. The equations of continuity and Navier-Stokes can be written as:
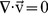
(1)

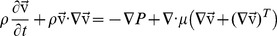
(2)where 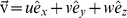, *P*, *ρ*, and *μ* represent velocity, pressure, blood mass density, and viscosity, respectively [25]–[27].

A FORTRAN program was used to implement the Galerkin FE method, which has been validated by theoretical solutions and experimental measurements [28]. Here, a mesh dependency was conducted such that the relative error in two consecutive mesh refinements was <1% for the maximum velocity of steady state flow with inlet flow velocity equal to the time-averaged velocity over a cardiac cycle. A total of approximately 600,000 linear tetrahedral finite elements (element edge of 0.3 mm) and 100,000 nodes were necessary to accurately mesh the computational domains. The backward method was used for the time integration. Three cardiac cycles were required to achieve convergence for the transient analysis. A constant time step was employed, where Δt = 0.00695 s with 121 total time step per cardiac cycle. Although blood is a suspension of particles, it behaves as a Newtonian flow in tubes with diameters >1 mm [29]. The experimentally-measured flow velocity wave (i.e., Fig. 5 in Ref. 3) was set as the boundary condition at the inlet of SVG, which had a blunt velocity profile as shown in Fig. 2. Since the proximal coronary artery had approximately zero flow due to occlusion, there was only a stress-free outlet boundary condition at the distal artery. The viscosity (*μ*) and density (*ρ*) of the solution were assumed as 4.0 cp and 1.06 g/cm^3^, respectively, to mimic blood flow with a hematocrit of about 45% in these arteries.

### Idealized Geometric Models

**Figure 3:**
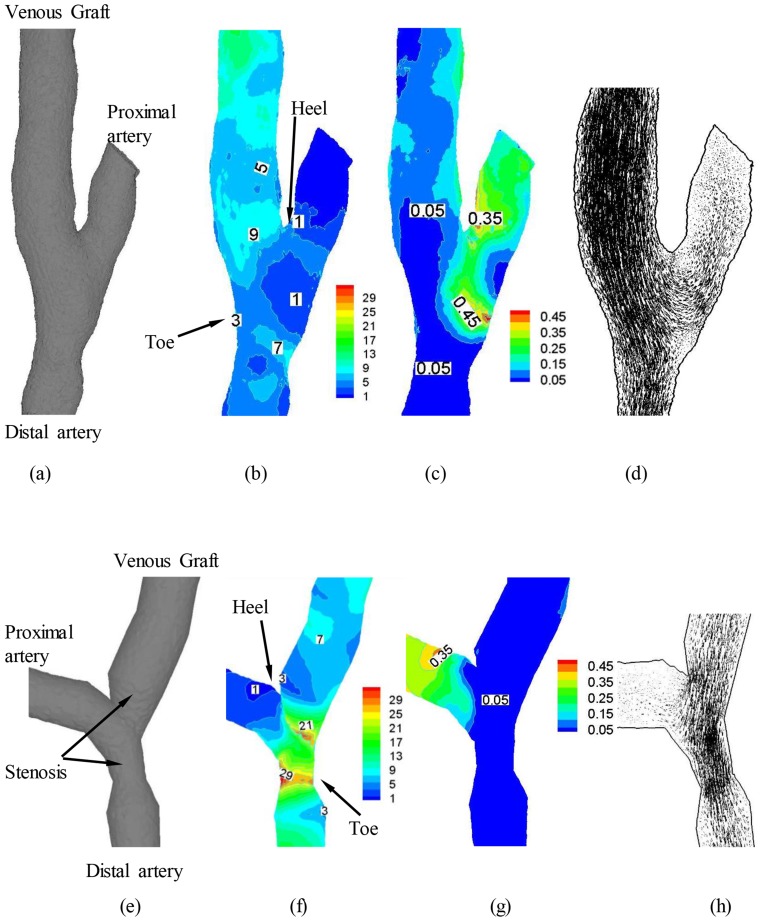
The anastomosis between SVG and coronary artery obtained from patient CTA with: (a) small anastomotic stenosis (area stenosis<20%) and the distribution of (b) time-averaged (over a cardiac cycle) WSS (Unit: Dynes⋅cm^−2^), (c) OSI and (d) time-averaged flow velocity (Unit: cm/s); (e) mild anastomotic stenosis (40%<area stenosis<60%) and the distribution of (f) time-averaged WSS (Unit: Dynes⋅cm^−2^), (g) OSI and (h) time-averaged flow velocity (Unit: cm/s).

To evaluate the relation between graft angle and graft-to-host cross-sectional area (CSA) ratio, we also constructed four idealized geometric models (graft-to-host CSA ratio 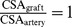 and graft angle θ = 45°; 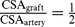 and θ = 45°; 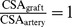 and θ = 85°; and 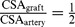 and θ = 85°, where 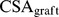 equal to the CSA of patient artery; e.g., from Fig. 3e).

### Data and Statistical Analysis

After the velocity and pressure of the blood flow were calculated, the hemodynamic parameters including WSS, OSI and WSSG were determined from the equations in the Appendix (Appendix S1). Moreover, a 2-sample student’s t Test (Microsoft Excel 2010) was used to compare the hemodynamic parameters between different anastomoses, where p value<0.05 represented a statistically significant difference.

## Results

Figure 2 shows a representative end-to-side SVG obtained from patient CTA. Figures 3a and 3e show anastomoses reconstructed from patient CTA with small stenosis (area stenosis<20%) and mild stenosis (40%<area stenosis<60%), respectively. Based on these morphometric data and the measured velocity waveform at the inlet of SVG, the flow simulations were performed to compute the distribution of hemodynamic parameters (e.g., WSS, WSSG and OSI) at the anastomosis between SVG and coronary artery. The mean Reynolds number (averaged over a cardiac cycle) and Womersley number varied in the range of 80–110 and 2–2.8, respectively, at the proximal site of SVG. Since peak Reynolds number <250 at the inlet of SVG and area stenosis <60%, transitional and turbulent flows did not occur. Figures 3b–d and Figures 3f–h show the distribution of time-averaged WSS (Unit: Dynes⋅cm^−2^), OSI, and time-averaged velocity (Unit: cm/s) at anastomoses with small and mild stenoses, respectively.

**Figure 4:**
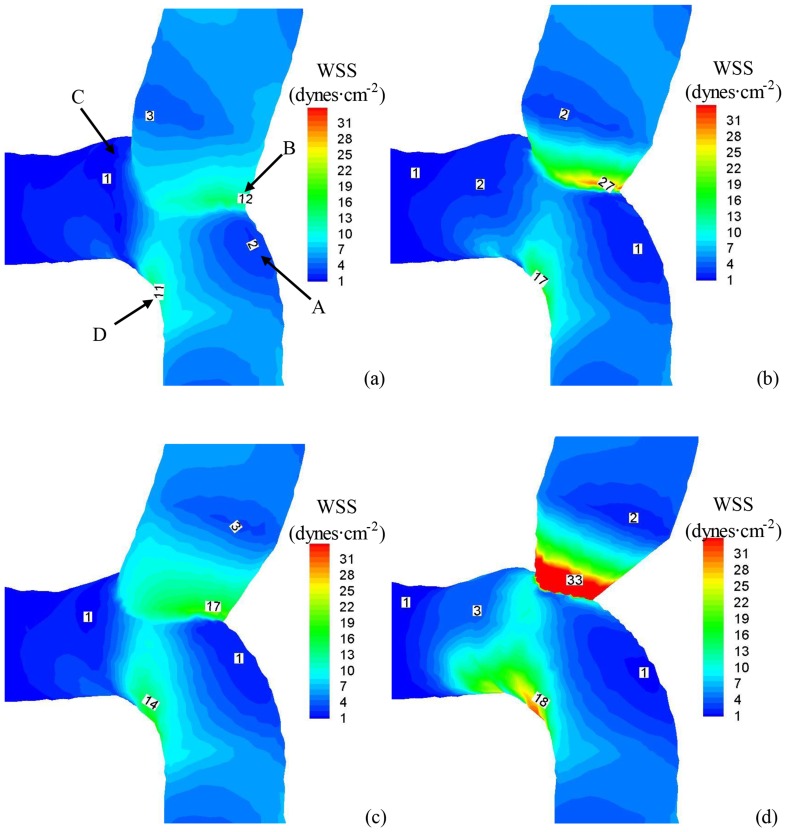
The distribution of time-averaged (over a cardiac cycle) WSS (Unit: Dynes⋅cm^−2^) at the anastomosis between SVG and coronary artery for geometrical models as: (a) 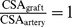 and θ = 45° (an idealized case, where 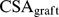 equal to the CSA of proximal artery in Fig. 3e); (b) 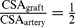 and θ = 45° (a simulated decrease of graft CSA); (c) 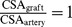 and θ = 85° (a simulated increase of graft angle); and (d) 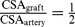 and θ = 85° (a simulated decrease of graft CSA as well as a simulated increase of graft angle). Region A refers to the toe region in coronary artery while Region B is the toe region in venous graft. Region C refers to the heel region. Region D represents coronary artery wall opposite to junction orifice.

**Figure 5:**
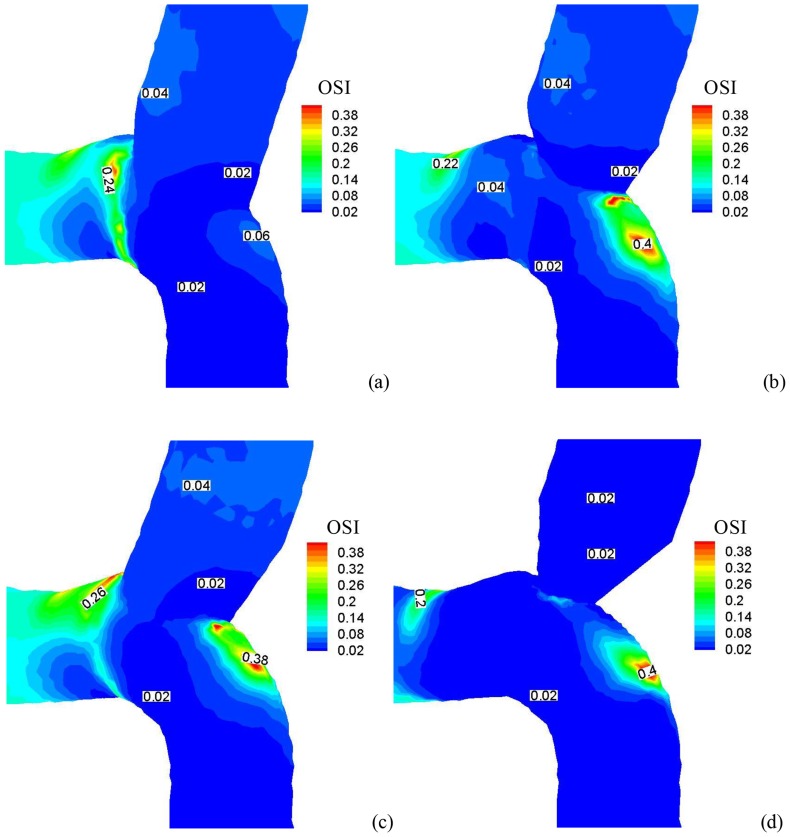
The distribution of OSI at the anastomosis between SVG and coronary artery in correspondence with Fig. 4.

**Figure 6:**
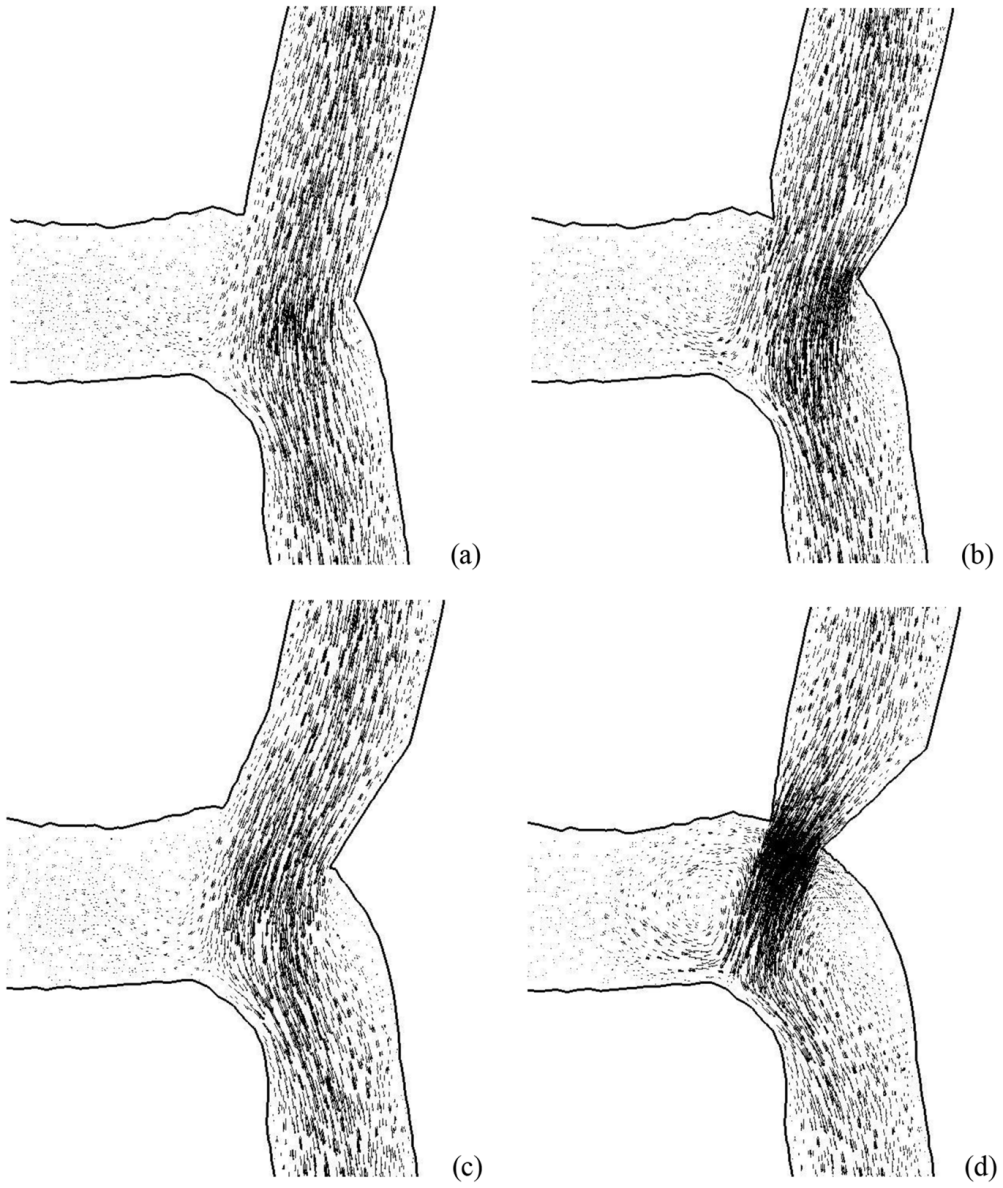
The distribution of time-averaged (over a cardiac cycle) flow velocity (Unit: cm/s) at the anastomosis between SVG and coronary artery in correspondence with Fig. 4.

Figures 4a–d show the distribution of time-averaged WSS (Unit: Dynes⋅cm^−2^) at the anastomosis between SVG and coronary artery for the four idealized models. Figures 5a–d show the distribution of OSI and Figures 6a–d show the distribution of time-averaged flow velocity (Unit: cm/s). Region A in Fig. 4a refers to the toe region in coronary artery (low WSS, high OSI, and low WSSG) while Region B is the toe region in venous graft (high WSS, low OSI, and high WSSG). Region C refers to the heel region (low WSS, high OSI, and low WSSG). Finally, Region D represents coronary artery wall opposite to junction orifice (high WSS, low OSI, and high WSSG).

**Figure 7:**
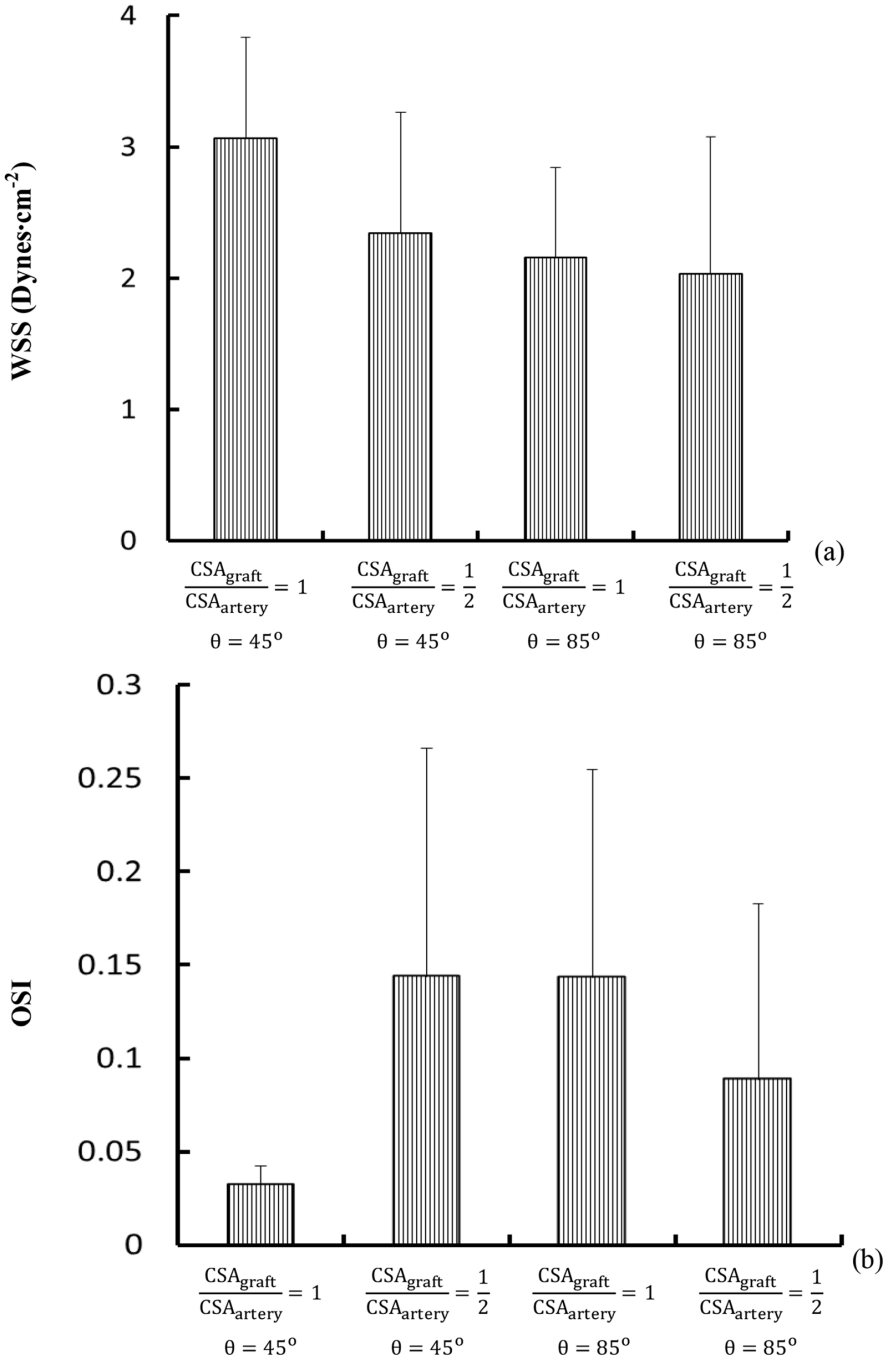
Mean ± SD (a) WSS and (b) OSI in Region A (averaged over all nodes in the region). Region A has surface area of 2.4, 6.8, 7.2, and 7.3^2^ for 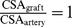 and θ = 45°; 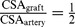 and θ = 45°; 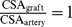 and θ = 85°; and 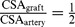 and θ = 85°, respectively.

**Figure 8:**
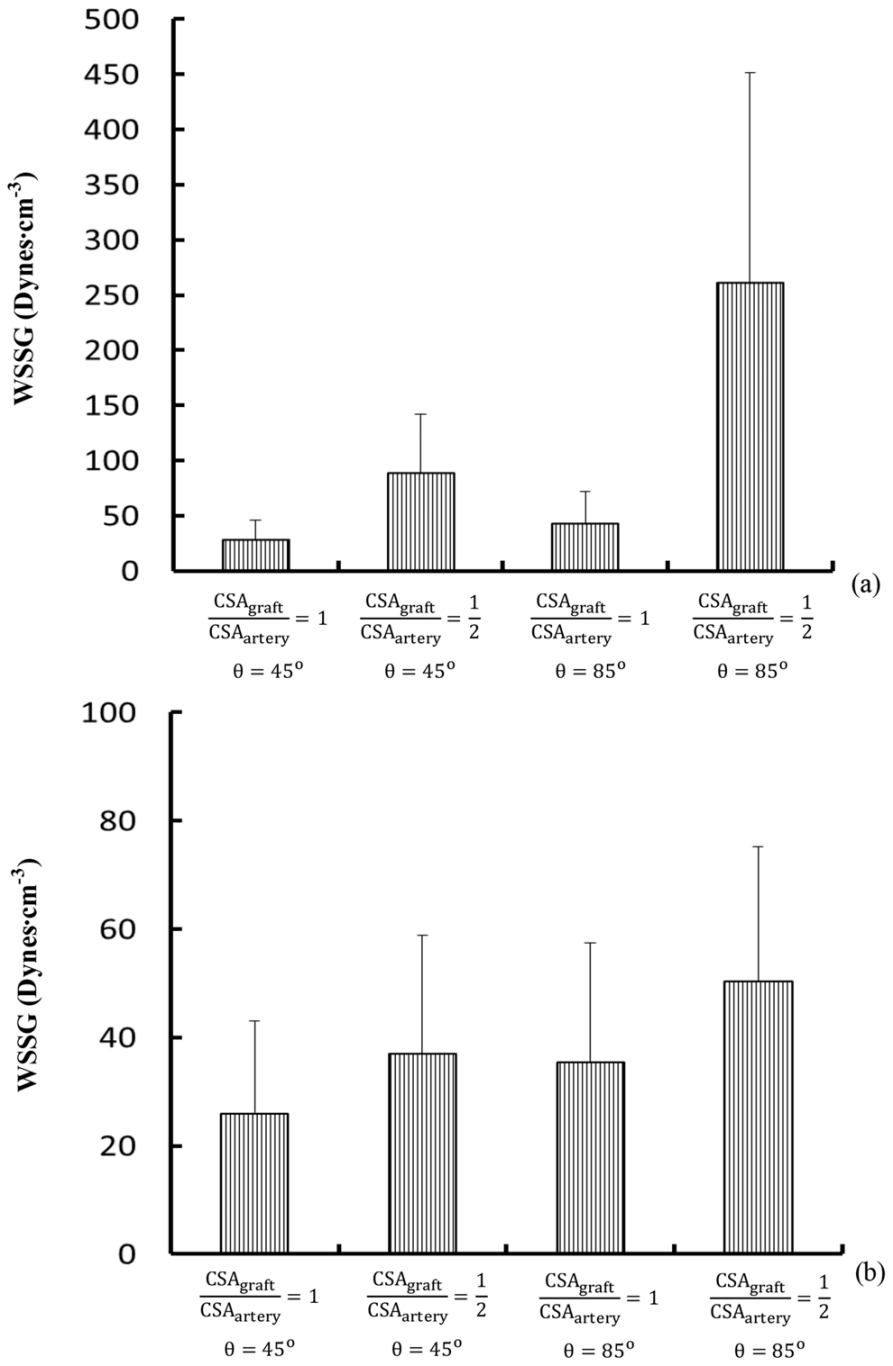
(a) Mean ± SD WSSG (Unit: Dynes⋅cm^−3^) in Region B (averaged over all nodes in the region), (b) Mean ± SD WSSG in Region D (averaged over all nodes in the region). Region B has surface area of 1.2, 2.8, 3.1, and 2.4 mm^2^ for 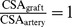 and θ = 45°; 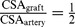 and θ = 45°; 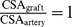 and θ = 85°; and 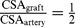 and θ = 85°, respectively. Region D has surface area of 2.4, 3.3, 4.2, and 4.3 mm^2^, accordingly.

Figures 7a and 7b show mean±SD values of WSS (Unit: Dynes⋅cm^−2^) and OSI, respectively, in Region A (averaged over all nodes in the region), where a decrease of graft-to-host CSA ratio (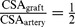 and θ = 45°) or an increase of graft angle (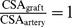 and θ = 85°) results in a 30% decrease of WSS and a four time increase of OSI at the toe region in coronary artery (p value<0.05). Figures 8a and 8b show mean±SD WSSG (Unit: Dynes⋅cm^−3^) in Regions B and D, respectively, where a decrease of graft-to-host CSA ratio as well as an increase of graft angle (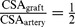 and θ = 85°) significantly increases WSSG at the toe region in SVG (by factor of ten, p value<0.05) and the coronary artery wall opposite to junction orifice (by factor of two, p value<0.05). Region A has surface area of 2.4, 6.8, 7.2, and 7.3 mm^2^ for 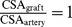 and θ = 45°; 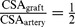 and θ = 45°; 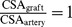 and θ = 85°; and 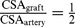 and θ = 85°, respectively. Accordingly, Region B has surface area of 1.2, 2.8, 3.1, or 2.4 mm^2^ and Region D has surface area of 2.4, 3.3, 4.2, or 4.3 mm^2^.

## Discussion

A mild anastomotic stenosis (area stenosis of 40–60%) enhances hemodynamic conditions (i.e., an increase of time-averaged WSS and a decrease of OSI inside the stenosis) albeit the proximal/distal sites to the stenosis show deteriorated hemodynamics. This suggests retarded atherosclerosis inside the stenosis, with enhanced atherosclerosis upstream and downstream of stenosis.

### Morphometry and Hemodynamics in the Long-term SVG

The reconstructed anastomoses showed mild stenoses in most patients for ≥10 years after operation, but relatively less in patients for >1 year and <6 years. These anastomotic stenoses were mainly attributed to atherosclerosis [30]. The short-term SVG occlusions due to thrombosis and intimal hyperplasia within the first year postoperatively were not included here because these have been substantially studied [4], [31], [32] and can be inhibited significantly by various drugs (Antiplatelet therapy, Beta blockers, Nitrates, etc.).

There were low time-averaged WSS (≤4 dynes⋅cm^−2^) and high OSI (≥0.15) at toe and heal regions of small anastomotic stenosis between SVG and coronary artery, as shown in Figs. 3b and 3c, respectively. The low WSS and high OSI were caused by the stagnated and reversed flows in Fig. 3d, similar to those at coronary bifurcations [24], [27]. On the other hand, the computational results showed that mild stenosis significantly altered the distribution of time-averaged WSS and OSI near the anastomosis in Figs. 3f and 3g. The interplay of decreased lumen CSA and increased flow velocity contributed to a significant increase of WSS (>15 dynes⋅cm^−2^) inside the stenosis given that 
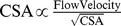
. The increased flow velocity also led to larger inertial force in the stenosis, which impeded flow reversal and decreased OSI (<0.02). The mild stenosis also resulted in a decrease of WSS (≤4 dynes⋅cm^−2^), but no significant increase of OSI at proximal and distal sites to the mild stenosis, as shown in Figs. 3f and 3g respectively.

### Implications for Long-term SVG Patency

As compared with intimal hyperplasia in subacute period (one to twelve months after CABG) [4], [33], atherosclerosis is the major risk factor for long-term SVG (>1 year) [30]. Abnormal flow pattern is a significant contributor to the progression of atherosclerosis [34], [35]. The mild stenosis, however, improves the hemodynamic conditions (Figs. 3a–d vs. Figs. 3e–h). An interesting analogy can be made in nature for ‘remodeling’ of rivers by flow. More than 400 years ago, Pan Quarter Tame (1521 to 1595) proposed a theory of ‘contracting water for sand discharge to keep river channels from rising in the lower reaches of the Yellow River’, which has recently been validated when mild narrow channels were selected [36], [37]. Similarly, the mild stenosis increases the flow velocity to prevent the progression of atherosclerosis inside it but does not significantly affect myocardial flow until the focal area stenosis reaches 75% or greater [38], [39]. This may help explain the decreased rate of graft failure as atherosclerosis stabilizes at long term (≥10 years) postoperatively [19], [20].

Moreover, the decrease in WSS at proximal and distal sites to the stenosis is mainly caused by the stagnated flows, as shown in Fig. 3h, which can also lead to the long residence time at those sites. This may be a significant risk factor for atherosclerotic expansion to become diffuse from a mild stenosis, which needs further studies in relation to SVG propensity for diffuse disease [19], [20] and plaque rupture [31].

### Effects of Anastomotic Geometry on Hemodynamics

The effect of geometry of anastomosis on arterial bypass graft patency has been investigated by many researchers [6], [40], [41]. The graft angle and the graft-to-host diameter/CSA ratio were two disputed subjects of those studies. There were different conclusions about the effects of graft angle on CABG patency, e.g., an optimal 45° angle [14], no correlation between intimal hyperplasia and graft angle [42], an optimal angle related to Reynolds number [15], and strong mixing for large angle [16]. Similar to the graft angle, experimental and numerical analysis of the graft-to-host diameter/CSA ratio also resulted in controversy, which included an optimal ratio of unity [43], an optimal diameter ratio of 1.6–2.1 [17], and no correlation between intimal hyperplasia and graft-to-host diameter/CSA ratio [18]. The relationship between the patency and graft angle and graft-to-host diameter/CSA ratio remains unknown.

In comparison with the anastomosis in Fig. 3e reconstructed from patient CTA for >1 year postoperatively, flow simulations were carried out in four idealized types of anastomoses (i.e., 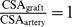 and θ = 45°; 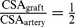 and θ = 45°; 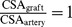 and θ = 85°; and 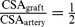 and θ = 85°) to investigate the relationship between flow patterns and graft angle and graft-to-host CSA ratio. The toe region in coronary artery (Region A in Fig. 4a) has low WSS and high OSI while the toe region in SVG (Region B) has high WSSG. The heel region (Region C) has low WSS and high OSI, but the coronary artery wall opposite to junction orifice (Region D) has high WSSG. A decrease of graft-to-host CSA ratio (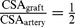 and θ = 45°) decreases WSS and increases OSI in Region A, similar to an increase of graft angle (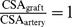 and θ = 85°). A decrease of graft-to-host CSA ratio as well as an increase of graft angle (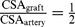 and θ = 85°) not only decrease WSS and increase OSI in Region A, but also increase WSSG significantly in Regions B and D. On the other hand, there is an approximate two-fold increase in surface areas of Regions A, B and D as the graft-to-host CSA ratio decreases or the graft angle increases. The heel region (Region C in Fig. 4a) has low WSS and high OSI due to the occluded proximal coronary artery regardless of the graft-to-host CSA ratio and graft angle.

An increase of graft-to-host CSA ratio or a decrease of graft angle improves hemodynamic conditions at the anastomosis between SVG and coronary artery which is consistent with previous studies [15]. Based on these findings, an optimal design of graft-to-host CSA ratio and graft angle was thought to improve the long-term patency by inhibiting intimal hyperplasia. The increased cyclic intramural tension in SVG due to a sudden increase of pressure, however, predominates intimal hyperplasia within the first year postoperatively [44], [45]. Drug inhibition of intimal hyperplasia has not improved the long-term CABG patency [46], [47]. In particular, the mild stenosis due to atherosclerosis significantly increases WSS (>15 dynes⋅cm^−2^) and decreases OSI (<0.02) and results in more uniform distribution of hemodynamic parameters inside the anastomosis in Figs. 3f–h as compared with those in Figs. 4–6. These findings make it necessary to reconsider the optimal graft design, particularly with respect to the relationship between hemodynamics and atherosclerosis as described above.

### Critique of Model

The flow velocity at the inlet of SVG was obtained from literature measurements [3]. The patient-specific velocity should be measured and used for numerical simulations in future studies. Furthermore, multiscale modeling with consideration of endothelial molecular processes that may contribute to stenosis is required in future studies. Finally, compliance mismatch is known to be an important risk factor for intimal hyperplasia and atherosclerosis in peripheral arterial bypass and arteriovenous grafts, particularly for prosthetic grafts. The compliance mismatch in CABG, however, is relatively smaller. The atherosclerosis formation in the anastomosis; e.g., Fig. 3, can further reduce the compliance mismatch. Hence, fluid-structure interaction is not needed in these simulations.

## Conclusion

Patient-specific mild anastomotic stenosis (area stenosis of 40–60%) increased WSS and decreased OSI inside the stenosis, but had a converse effect on WSS at proximal and distal sites to the stenosis. The favorable hemodynamics inside a mild stenosis may be a stabilizing factor to decrease the rate of graft failure despite the initial designs with no optimal graft-to-host CSA ratios or graft angles. Moreover, the changes at proximal and distal sites to the stenosis may contribute to diffuse expansion of atherosclerosis from a mild stenosis which could ultimately affect the long-term graft patency. To bolster the patient-specific conclusions, we also carried out the flow simulations in four idealized types of anastomoses and found that an increase of graft-to-host CSA ratio or a decrease of graft angle, between SVG and coronary artery, improved hemodynamic conditions.

## Supporting Information

Appendix S1 **Appendix.**
(DOCX)
