# Supplementary material for: Mild Anastomotic Stenosis in Patient-Specific CABG Model May Enhance Graft Patency: A New Hypothesis
Source: PLoS One. 2013 Sep 13;8(9):e73769. doi: 10.1371/journal.pone.0073769 (PMC3772875; doi:10.1371/journal.pone.0073769)
Supplement: Appendix S1 — Appendix. (DOCX) [file pone.0073769.s001.docx]

# Appendix S1

## Hemodynamic Parameters

Based on the computed flow filed, Reynolds (Re) and Womersley ($\alpha$) numbers are determined, which are defined, respectively, as follows:

$\mathrm{Re}=\frac{\rho V\cdot D}{\mu}$ [A1]

$\alpha=\frac{D}{2}\sqrt{\frac{\omega\rho}{\mu}}$ [A2]

Where $V$, *D*, $\omega$, $\rho$, and $\mu$ represent the velocity at the inlet of CABG, diameter of CABG, angular frequency of beating hearts, blood mass density, and viscosity, respectively. When $V$ refers to the time-averaged or peak velocity, there is mean or peak Reynolds number.

At any point of 3D FE model, the stress can be represented as a nine-component tensor (), which can be written as follows:

[A3]

where is the shear rate tensor. The stress on the wall, its normal component, and its two tangential components can be written as, respectively:

, , and [A4]

where $\text{n}$, $\text{t}_{1}$, and $\text{t}_{2}$ are the unit vector in the normal and two tangential directions, respectively. The shear component of $\vec{\tau}$ has the vector form:

[A5]

Equation [A5] is used to calculate WSS, which has the magnitude: . The time-averaged WSS over a cardiac cycle (*T*) can be written as follows:

[A6]

The present time-averaged OSI can be written as follows:

[A7]

Equations [A5-A7] were used to calculate the WSS and OSI in the FE model. The computation of WSSG has been described previously [23].
